# Efficacy and safety of peripherally-restricted κ-opioid receptor agonist-HSK21542 for postoperative analgesia in patients undergoing laparoscopic abdominal surgery: a randomized, placebo-controlled phase 2 trial

**DOI:** 10.3389/fmed.2025.1604790

**Published:** 2025-07-24

**Authors:** Yinbo Zhong, Haiying Wang, Min Yan, Mengchang Yang, Jiaqiang Zhang, Ling Nan, Zhiping Wang, Jianjun Yang, Jinglei Wu, Qulian Guo, Xiaoling Hu, Hongmeng Xu, Qiang Xu, Dongxin Wang

**Affiliations:** ^1^Department of Anesthesiology, The Second Affiliated Hospital, Zhejiang University School of Medicine, Hangzhou, China; ^2^Department of Anesthesiology, Sichuan Provincial People’s Hospital, School of Medicine, University of Electronic Science and Technology of China, Chengdu, China; ^3^Department of Anesthesiology, Pain and Perioperative Medicine, Henan Provincial People’s Hospital, Zhengzhou, China; ^4^Department of Anesthesiology, The First Bethune Hospital of Jilin University, Changchun, China; ^5^Department of Anesthesiology, The Affiliated Hospital of Xuzhou Medical University, Xuzhou, China; ^6^Department of Anesthesiology, Pain and Perioperative Medicine, The First Affiliated Hospital of Zhengzhou University, Zhengzhou, China; ^7^Department of Anesthesiology, Liuzhou People’s Hospital Affiliated to Guangxi Medical University, Liuzhou, China; ^8^Department of Anesthesiology, Xiangya Hospital, Central South University, Changsha, China; ^9^Department of Anesthesiology, The First Affiliated Hospital of University of South China, Hengyang, China; ^10^Department of Anesthesiology, The Fourth Hospital of Hebei Medical University, Shijiazhuang, China; ^11^Department of Anesthesiology, Union Hospital, Tongji Medical College, Huazhong University of Science and Technology, Wuhan, China; ^12^Department of Anesthesiology, Peking University First Hospital, Beijing, China

**Keywords:** κ-opioid receptor agonists, postoperative analgesia, laparoscopic abdominal surgery, HSK21542, time-weighted summed pain intensity difference

## Abstract

**Background:**

This phase 2 trial comprised dose exploration (stage 1) and dose confirmation stages (stage 2) to determine the safety and efficacy of HSK21542 in patients undergoing laparoscopic abdominal surgery.

**Methods:**

In stage 1, patients were randomly allocated at a ratio of 4:1 (12 to receive HSK21542, 3 to receive placebo) to 4 ascending dose groups in a sequential manner (group 1: preoperative HSK21542-0.4 μg/kg (or placebo) + HSK21542-0.2 μg/kg (or placebo) at postoperative 0 h, 8 h and 16 h; group 2: preoperative HSK21542-1.0 μg/kg (or placebo) + HSK21542-0.5 μg/kg (or placebo) at postoperative 0 h, 8 h and 16 h; groups 3 and 4: HSK21542-0.5 μg/kg or HSK21542-1.0 μg/kg (or placebo) at postoperative 0 h, 8 h and 16 h). In stage 2, patients received HSK21542-0.5 μg/kg, HSK21542-1.0 μg/kg or placebo postoperatively at 0 h, 8 h and 16 h in a 1:1:1 ratio. The primary endpoints in stage 1 were the safety outcomes including the incidence and severity of treatment-emergent adverse events (TEAEs) while the primary endpoint of stage 2 was the time-weighted summed pain intensity differences over 24 h (SPID_0–24__*h*_).

**Results:**

Stage 1 enrolled 63 patients and 57 completed the trial, while 61 patients were enrolled in stage 2, and 60 completed the trial. The most common TEAEs were fever (22.9% vs. 41.7%), nausea (25.0% vs. 33.3%) and vomiting (22.9% vs. 25.0%) in the HSK21542 and placebo groups in stage 1. HSK21542 doses of 0.5 μg/kg and 1.0 μg/kg administered postoperatively were recommended for the subsequent stage 2. The pooled results revealed a slightly lower SPID_0–24__*h*_ in HSK21542-1.0 μg/kg group (−1,679.8 ± 2,284.3 scores × min) than those in HSK21542-0.5 μg/kg (−1,499.4 ± 2,487.2 scores × min) and placebo groups (−435.2 ± 2,852.9 scores × min; *P* = 0.114). A significantly higher least squares mean difference of pain intensity differences (PID) was found in HSK21542-1.0 μg/kg group compared to the placebo (*P* = 0.020).

**Conclusion:**

HSK21542 at all dose regimens demonstrated well tolerability and safety comparable to that of the placebo among patients undergoing laparoscopic abdominal surgery in the phase 2 trial. The dosing regimen of HSK21542-1.0 μg/kg administered postoperatively at 0 h, 8 h and 16 h exhibited an acceptable efficacy, warranting its recommendation for further phase 3 trial.

**Clinical trial registration:**

https://clinicaltrials.gov/, identifier NCT04424251.

## 1 Introduction

Laparoscopic surgery has obvious advantages such as smaller incisions, less blood loss, a shorter hospitalization duration, faster recovery and a lower risk of infection and complications when applied to less complex surgery compared with conventional open surgery (1). However, the incidence and severity of postoperative pain after laparoscopic surgery were not inferior to those after open surgery, especially in the early postoperative period (2). Therefore, postoperative pain management is also critical for laparoscopic surgery, requiring the use of opioids and non-opioids analgesics. Previous studies have also reported the non-opioid postoperative analgesia approaches to reduce the opioids-related adverse effects (3). However, opioid analgesics are still the main drugs commonly used for postoperative analgesia after laparoscopic surgery, which exert analgesic effects by stimulating opioid receptors in the peripheral and central nervous systems (CNS). Despite the strong analgesic effects of opioid analgesics, their treatment is often accompanied by severe side effects, such as respiratory depression, drug addiction, constipation, nausea, immune suppression and tolerance (4). At present, the confirmed opioid receptors include μ, κ, δ and nociception/orphanin FQ, among which μ, κ and δ receptors are closely related to postoperative analgesia (5). The representative drugs, which are early central κ-opioid receptor (KOR) agonists, include U-50488, ICI-199441, spiradoline (U-6266E) and enadoline, that produce good analgesic activity without morphine-like side effects (i.e., respiratory depression, constipation, addiction and tolerance), and are able to antagonize the drug dependence caused by μ receptor activation (6). However, the results of clinical studies on spiradoline and enadoline were disappointing, and CNS-mediated side effects such as sedation, anxiety and diuresis gradually became apparent (7, 8). Alternatively, the activation of peripheral KOR may also exert analgesic effects by attenuating the excitability of peripheral pain reactive endings and weakening action potential transmission in sensory neurons (9). Therefore, much research has focused on the design of novel, selective, peripherally-restricted KOR agonists, which can only act in the periphery and rarely cross the blood-brain barrier (BBB), thus not only maintaining their high agonist activity at KOR, but also avoiding the inherent side effects of traditional central selective KOR agonists.

HSK21542 is a novel, selective, peripherally-restricted KOR agonist, which is expected to be used for postoperative pain, as it rarely crosses the BBB, leading to a lower incidence of side effects in the CNS, such as hallucination, addiction and respiratory depression, being a candidate drug for treatment of both pain and pruritus (10). Preclinical studies have confirmed that HSK21542 has a favorable safety profile in rats and rabbits, with only minor effects on embryonal-fetal developmental outcomes (11), and had no potential for abuse in rats (12). A study designed to investigate the pharmacokinetics, mass balance, metabolism and excretion of HSK21542 showed that HSK21542 is a short-chain compound that is minimally metabolized in the body and is mainly excreted by the kidney (13). Another study that developed a physiologically-based pharmacokinetic (PBPK) model of HSK21542 indicated that systemic exposure to HSK21542 was increased in patients with renal insufficiency, hepatic insufficiency and the elderly, but decreased in pediatric patients, which provided guidance for the dosage of HSK21542 in special populations (14). In the first-in-human phase 1 study, a single bolus of HSK21542 was found to be well tolerated in the range 0.2∼3.375 μg/kg as a 15-min constant-dose intravenous (i.v.) infusion (15). Moreover, the mean C_*max*_ following the HSK21542-0.2 and 1.0 μg/kg was slightly higher with a 2-min infusion duration than with a 15-min infusion duration, but the AUC was comparable. Another multiple ascending dose study also confirmed a promising itching effect of HSK21542 for hemodialysis patients (16).

Therefore, a multicenter, randomized, double-blind, two-stage phase 2 trial that comprised a dose exploration stage (stage 1) and a dose-confirmation stage (stage 2) was conducted to evaluate the efficacy and safety of HSK21542 for postoperative analgesia in patients undergoing elective laparoscopic abdominal surgery. The primary objective of the stage 1 study was to assess the safety and tolerability of HSK21542 for postoperative analgesia, followed by efficacy, pharmacodynamics (PD) and PK profiles to determine the recommended dose and frequency of HSK21542 for a subsequent stage 2 study. The objective of the stage 2 study was to evaluate the efficacy, safety and PK profiles of HSK21542 for postoperative analgesia in patients in a larger cohort size.

## 2 Materials and methods

### 2.1 Trial design and patients

It was a multicenter, randomized, double-blind, placebo-controlled, two-stage phase 2 clinical trial conducted in 14 hospitals across China that comprised a dose exploration stage (stage 1) and a dose-confirmation stage (stage 2). In stage 1, eligible patients were randomly allocated at a ratio of 4:1 (12 to receive HSK21542, 3 to receive placebo) to 4 ascending dose groups in a sequential manner (group 1: preoperative 0.4 μg/kg + 0.2 μg/kg at postoperative 0 h, 8 h and 16 h; group 2: preoperative 1.0 μg/kg + 0.5 μg/kg at postoperative 0 h, 8 h and 16 h; group 3: 0.5 μg/kg at postoperative 0 h, 8 h and 16 h; group 4: 1.0 μg/kg at postoperative 0 h, 8 h and 16 h. In stage 2, eligible patients were randomly assigned to receive HSK21542-0.5 μg/kg, HSK21542-1.0 μg/kg or placebo at postoperative 0 h, 8 h and 16 h in a 1:1:1 ratio. The studies involving humans were approved by ethical committee of the Second Affiliated Hospital Zhejiang University School of Medicine (approval number: 2020-674) and all other participating centers. The studies were conducted in accordance with the local legislation and institutional requirements. The participants provided their written informed consent to participate in this study. The trial was prospectively registered with clinicaltrials.gov on June 3, 2020 under identifier NCT04424251.

This trial included patients aged 18 to 70 years (inclusive) with American Society of Anesthesiologists (ASA) grades I-II, a body mass index (BMI) of 18 to 40 kg/m^2^ and those expected to undergo elective laparoscopic surgery for 1–5 h (inclusive) under general anesthesia. The main exclusion criteria for the trial were: a history of allergy to opioid drugs, with symptoms such as urticaria; allergy to the intraoperative anesthetic drugs specified in the protocol; a history of cardiovascular, respiratory, neurological or psychiatric disease; respiratory management risk identified prior to screening; patients with serum creatinine > 1.5 × upper limit of normal (ULN); patients who had undergone major surgery within the past 3 months that was judged by the investigator to impact on the postoperative pain assessment; patients with a pulse oxygen saturation < 92% without supplemental oxygen at screening; patients who had donated blood or lost blood > 400 mL within the past 3 months; and patients with a history of drug abuse, substance use and/or alcohol abuse within the past 3 months. Patients were also excluded if they had been given an analgesic agent with an uncertain half-life within 14 days before randomization or if they been given an analgesic drug before randomization and had received the last administration < 5 half-lives before randomization. Detailed inclusion and exclusion criteria are presented in Supplementary Appendix 1.

### 2.2 Randomization and masking

This trial was conducted through an interactive Web response system (IWRS) using a block randomization method, and eligible patients were given randomization and corresponding medication numbers, where the medication number for each patient represented the corresponding treatment arm. In stage 1, 60 eligible patients were randomly allocated at a ratio of 4:1 (12 to receive HSK21542, 3 to receive placebo) into 4 ascending dose groups and dose exploration of the first 3 groups was carried out step by step according to the sequence, that is, the assessment of the next dose group was carried out only after administration of the previous dose group was completed and the safety assessment qualified (3 days after administration). In stage 2, 60 eligible patients were randomly assigned to HSK21542-0.5 μg/kg, HSK21542-1.0 μg/kg or placebo groups in a 1:1:1 ratio. The trial adopted a double-blind design, in which patients, investigators and assessors were blinded to the treatment-group assignments, whereas the unblinded statistician was responsible for the preparation and management of the IWRS.

### 2.3 Study procedures

**Stage 1:** Patients in groups 1 and 2 received a single i.v. injection of HSK21542-0.4 μg/kg or HSK21542-1.0 μg/kg, respectively, or a corresponding placebo after skin incision (± 10 min). Remifentanil was discontinued immediately after skin closure (i.e., the last stitch was made) and a top-up dose of sufentanil (0.15 μg/kg) was administered i.v. 15–30 min before the end of the skin closure, with a total dosage of < 10 μg. Patients in groups 1–4 received the first dose of the corresponding HSK21542 (0.2 μg/kg, 0.5 μg/kg, 0.5 μg/kg, 1.0 μg/kg) or placebo i.v. within 10 min after surgery, and the end time of the first dose after surgery was recorded as 0 min. Subsequently, the corresponding doses of HSK21542 (0.2 μg/kg, 0.5 μg/kg, 0.5 μg/kg, 1.0 μg/kg) or placebo were administered i.v. 8 h (± 15 min) and 16 h (± 15 min) after the operation. The initial dose selection of 0.2 μg/kg, 0.5 μg/kg and 1.0 μg/kg in stage 1 was mainly based on the phase 1 trial of HSK21542, and also took into account the phase 3 trial design of difelikefalin (NCT02542384). From the results of the phase 1 trial, a single bolus of HSK21542 was found to be well tolerated in the range 0.2∼ 3.375 μg/kg as a 15-min constant-dose i.v. infusion (15). Moreover, mean C_*max*_ following the HSK21542-0.2 and 1.0 μg/kg was slightly higher with a 2-min infusion duration than with a 15-min infusion duration, but the AUC was comparable. Considering that HSK21542 was explored in healthy subjects only as a single bolus in the phase 1 study, and multiple doses of HSK21542 were used for the first time in patients undergoing laparoscopic abdominal surgery in the present phase 2 trial, 0.2 μg/kg was chosen as an initial dose for dose escalation in accordance with the phase 1 trial. In addition, 0.5 and 1.0 μg/kg of HSK21542 were chosen on the basis of the difelikefalin dose used in the phase 3 clinical trial. Considering that the difelikefalin was administered as an i.v. bolus 2 × loading dose 1-h prior to anesthetic induction, thus preoperative 0.4 μg/kg and 1.0 μg/kg of HSK21542 were used for groups 1 and 2 in stage 1; 0.2 and 0.5 μg/kg of HSK21542 were administered postoperatively.

**Stage 2:** Patients in the 3 groups only received the corresponding dose of HSK21542 (0.5 μg/kg, 1.0 μg/kg) or placebo i.v. within 30 to 60 min after incision sutures, 8 h (± 15 min) and 16 h (± 15 min) after the operation. Other perioperative management methods were the same as for stage 1.

The numeric rating scale (NRS) was used to assess postoperative pain at rest (such as sitting or lying down) at 10 min before the first postoperative administration of experimental drugs, 15 min (± 5 min), 30 min (± 5 min), 1 h (± 5 min), 2 h (± 15 min), 4 h (± 15 min), 8 h (30 min before the second postoperative dose), 12 h (± 30 min), 16 h (30 min before the last postoperative dose), and 24 h (± 1 h) after the first postoperative administration. If the NRS at rest was ≥ 4 at any time point after the first postoperative administration of HSK21542, or if patients complained of pain at any time point and the NRS at rest was ≥ 4, a morphine injection was given for remedial treatment. The time from NRS assessment to actual morphine injection was ≤ 30 min, with an initial morphine dose of 3 mg, followed by a further morphine dose as required. In addition, the interval between the twice morphine remedial treatment was ≥ 2 h, and the interval between the rescue drug and the HSK21542 dose was ≥ 1 h. Anti-emetic prophylaxis was prohibited during the study procedure, but investigators could administer anti-emetics on the basis of the degree of nausea and vomiting after the first postoperative dose of HSK21542, and the dose of anti-emetics administered was recorded. Tropisetron was the first-choice treatment for postoperative nausea and vomiting, with an initial dose of 2.5 mg, followed by an additional dose if required, not exceeding 10 mg within 24 h. If tropisetron was not effective, investigators could use other drugs according to good clinical practice.

### 2.4 Endpoints

**Stage 1:** The primary objective of the stage 1 trial was to assess safety and tolerability, followed by efficacy outcomes, PD and PK characteristics, in order to establish the recommended dose and frequency of HSK21542 to be used subsequently in the stage 2 trial. Therefore, the primary endpoints in stage 1 were the safety outcomes including the incidence and severity of treatment-emergent AEs (TEAEs), vital signs (respiratory rate, heart rate, systolic and diastolic blood pressure), pulse oxygen saturation (SpO_2_), 12-lead electrocardiogram (ECG) results, laboratory indicators, cumulative dosage and the proportion of anti-emetic drugs used within 0–24 h after the first postoperative administration of the experimental drugs. The secondary endpoints for the stage 1 trial were efficacy outcomes (same as for stage 2), and measurements of HSK21542 plasma concentrations and serum prolactin concentrations. The assessment methods for AEs and other safety indicators, plasma concentrations of HSK21542 and prolactin are presented in Supplementary Appendix 2.

**Stage 2:** The primary endpoint was the time-weighted summed pain intensity differences over 24 h (SPID_0–24__*h*_) after the first postoperative administration of experimental drugs. The secondary endpoints included SPID_0–12__*h*_, pain intensity differences (PID) at each time point, the proportion of patients with NRS ≤ 3, the cumulative dosage of morphine injections over 12 h and 24 h, the duration of analgesia, the satisfaction rating of patients and physicians, safety (same as in stage 1) and plasma concentration analyses.

### 2.5 Statistical analysis

All statistical analyses were performed using SAS ver. 9.4 software. For continuous variables, the mean ± standard deviation (SD), median with range (minimum to maximum) are given, and for categorical variables numbers and percentages are presented. Statistical significance was defined as a bidirectional alpha level of *P* < 0.05. However, since this trial was an exploratory study, and the sample size was specified in advance rather than determined based on power calculations, all *P*-values were exploratory. The definitions of the analysis set are listed in Supplementary Appendix 3.

For primary efficacy outcomes, SPID was compared between groups using analysis of variance (ANOVA). PID at each time point was compared using ANOVA, and another mixed model repeated measures (MMRM) method was also employed to support the analysis, with the baseline pain score, treatment, time point and interaction between treatment and time points as fixed effects, and patients as random effects. For the remaining efficacy outcomes, ANOVA or a non-parametric test (i.e., Kruskal-Wallis) was used to compare continuous variables between groups, and chi-squared or Fisher’s exact tests to compare categorical variables between groups.

The missing data for efficacy and safety outcomes were not imputed. However, for patients who had been given a rescue medication, such as morphine, within 24 h after the first dose of HSK21542 or placebo, the NRS score was corrected accordingly. If the patient was given one or more morphine rescue doses, and the interval between 2 consecutive rescue doses was > 6 h, the NRS score at the planned time point within 6 h was carried forward using the last NRS score before the current rescue medication, and the data after 6 h still used the actual NRS score. When > 1 rescue dose of morphine was administered but the interval between two consecutive morphine rescue doses was < 6 h, the NRS score between each consecutive dose was carried forward to the last NRS score before the previous rescue dose, and correction of the NRS score for the subsequent rescue dose was performed according to the principle described above.

## 3 Results

### 3.1 Enrollment of patients and their baseline characteristics

From July 2, 2020 to November 16, 2020, a total of 70 patients were screened in stage 1, and of 63 randomized patients, 60 received the experimental drugs (HSK21542 or placebo), with 12 in the placebo group and 48 in the HSK21542 dose groups (group 1: *n* = 12; group 2: *n* = 12; group 3: *n* = 12; group 4: *n* = 12). Finally, 57 patients completed the trial, with 12 in the placebo group and 45 in the HSK21542 dose groups (group 1: *n* = 10; group 2: *n* = 12; group 3: *n* = 12; group 4: *n* = 11). Therefore, all 60 patients who received the experimental drugs were enrolled in the safety set (SS) and PD analysis set (PDS). According to the definition of analysis set, 59 patients with available efficacy data were included in the full analysis set (FAS), and 45 patients with available plasma concentration were included in the PK analysis set (PKS).

**FIGURE 1:**
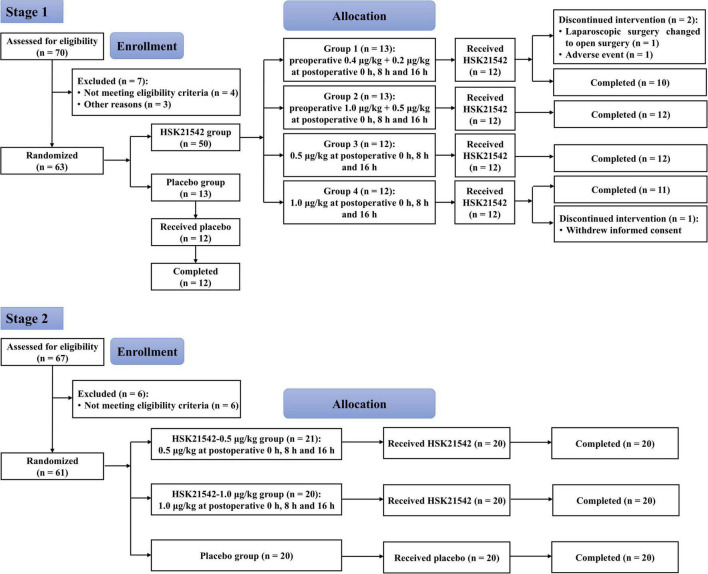
Disposition flow diagrams of enrolled patients in stages 1 and 2.

From October 26, 2020 to December 7, 2020, a total of 67 patients were screened in stage 2, with 61 patients enrolled for randomization. Of the 61 patients, 1 who did not receive HSK21542 in the HSK21542-0.5 μg/kg group withdrew early from the trial, and the remaining 60 (20 in each group) completed the trial (Figure 1). Finally, 60 patients were enrolled in the safety set (SS) and FAS, and 40 enrolled in PKS.

Overall, the demographic and baseline characteristics of the patients in the placebo and HSK21542 groups were generally similar (Table 1). All patients were female and ASA grade I-II, with a median age of 49 years in stage 1 and stage 2. All enrolled patients underwent laparoscopic hysterectomy in stage 1 (100%) and stage 2 (100%). In addition to the laparoscopic hysterectomy, some enrolled patients also underwent other laparoscopic abdominal procedures, including laparoscopic salpingectomy, pelvic lymphadenectomy and lysis of pelvic adhesions. The vast majority of patients had a history of leiomyoma of the uterus (57.6%), followed by adenomyosis (27.1%) and ovarian cysts (22.0%) in stage 1. Stage 2 mainly included patients with a history of leiomyoma of the uterus (63.3%), pelvic adhesions (50.0%) and adenomyosis (26.7%). The mean (± SD) NRS was comparable between the HSK21542 dose groups and placebo group in stage 1 (3.8 ± 2.3 vs. 2.7 ± 2.0 vs. 2.4 ± 1.2 vs. 3.4 ± 1.1 vs. 2.4 ± 1.6, *P* = 0.252) and stage 2 (3.2 ± 1.8 vs. 2.9 ± 1.6 vs. 2.5 ± 1.9, *P* = 0.363).

**TABLE 1:** Demographic and baseline characteristics of enrolled patients in stage 1 and stage 2 (FAS).

Variables	Stage 1					Stage 2		
	Placebo group (*n* = 12)	HSK21542 group (*n* = 47)				Placebo group (*n* = 20)	HSK21542 group (*n* = 40)	
		Group 1 (*n* = 11)	Group 2 (*n* = 12)	Group 3 (*n* = 12)	Group 4 (*n* = 12)		0.5 μg/kg (*n* = 20)	1.0 μg/kg (*n* = 20)
**Age (years)**								
Mean (SD)	46.1 (11.3)	49.5 (6.3)	48.8 (8.4)	48.7 (8.2)	47.1 (7.2)	51.0 (6.2)	48.2 (5.8)	49.1 (8.2)
Median (range)	44.0 (31–61)	49.0 (36–59)	49.5 (30–60)	48.0 (36–68)	48.5 (36–61)	50.0 (44–68)	47.0 (40–65)	49.0 (31–68)
**Age range, *n* (%)**								
18–40 years	5 (41.7)	1 (9.1)	2 (16.7)	2 (16.7)	3 (25.0)	0	1 (5.0)	3 (15.0)
41–65 years	7 (58.3)	10 (90.9)	10 (83.3)	9 (75.0)	9 (75.0)	19 (95.0)	19 (95.0)	16 (80.0)
> 65 years	0	0	0	1 (8.3)	0	1 (5.0)	0	1 (5.0)
Female, *n* (%)	12 (100)	11 (100)	12 (100)	12 (100)	12 (100)	20 (100)	20 (100)	20 (100)
**BMI (kg/m^2^)**								
Mean (SD)	24.5 (3.0)	24.0 (2.0)	23.1 (2.2)	23.1 (2.6)	23.4 (3.2)	24.4 (3.7)	24.0 (2.9)	26.0 (3.2)
Median (range)	24.3 (18.9–28.9)	23.5 (21.8–27.5)	23.4 (19.6–26.4)	23.2 (18.7–28.0)	23.4 (19.3–31.2)	23.6 (19.1–34.5)	24.1 (18.1–30.2)	25.5 (21.0–32.0)
**ASA classifications, *n* (%)**								
Stage I	1 (8.3)	0	1 (8.3)	2 (16.7)	4 (33.3)	9 (45.0)	8 (40.0)	5 (25.0)
Stage II	11 (91.7)	11 (100)	11 (91.7)	10 (83.3)	8 (66.7)	11 (55.0)	12 (60.0)	15 (75.0)
**Baseline NRS score**								
Mean (SD)	2.4 (1.6)	3.8 (2.3)	2.7 (2.0)	2.4 (1.2)	3.4 (1.1)	2.5 (1.9)	3.2 (1.8)	2.9 (1.6)
Median (range)	2.5 (0–5)	4.0 (1–8)	2.5 (0–6)	2.0 (1–4)	3.5 (2–5)	2.5 (0–8)	3.0 (1–8)	3.0 (0–5)
**Medical history (> 20%), *n* (%)**								
Leiomyoma of uterus	6 (50.0)	9 (81.8)	10 (83.3)	6 (50.0)	3 (25.0)	13 (65.0)	15 (75.0)	10 (50.0)
Adenomyosis	2 (16.7)	5 (45.5)	4 (33.3)	3 (25.0)	2 (16.7)	2 (10.0)	8 (40.0)	6 (30.0)
Ovarian cyst	2 (16.7)	3 (27.3)	3 (25.0)	4 (33.3)	1 (8.3)	1 (5.0)	4 (20.0)	6 (30.0)
Anemia	3 (25.0)	2 (18.2)	1 (8.3)	4 (33.3)	2 (16.7)	4 (20.0)	4 (20.0)	4 (20.0)
Cervicitis	1 (8.3)	3 (27.3)	2 (16.7)	2 (16.7)	3 (25.0)	4 (20.0)	3 (15.0)	0
Pelvic adhesions	1 (8.3)	1 (9.1)	1 (8.3)	4 (33.3)	2 (16.7)	11 (55.0)	11 (55.0)	8 (40.0)
Hypertension	2 (16.7)	1 (9.1)	2 (16.7)	2 (16.7)	0	5 (25.0)	4 (20.0)	2 (10.0)
Uterine polyp	1 (8.3)	1 (9.1)	0	3 (25.0)	1 (8.3)	2 (10.0)	4 (20.0)	3 (15.0)

For HSK21542 dose groups in stage 1: group 1: preoperative 0.4 μg/kg + 0.2 μg/kg at postoperative 0 h, 8 h and 16 h; group 2: preoperative 1.0 μg/kg + 0.5 μg/kg at postoperative 0 h, 8 h and 16 h; group 3: 0.5 μg/kg at postoperative 0 h, 8 h and 16 h; group 4: 1.0 μg/kg at postoperative 0 h, 8 h and 16 h. Stage 2: HSK21542-0.5 μg/kg or HSK21542-1.0 μg/kg were administered postoperatively at 0 h, 8 h and 16 h. BMI, body mass index; FAS, full analysis set; NRS, numerical rating scale; SD, standard deviation.

### 3.2 Safety

For SS in stage 1, 37 (77.1%) patients in the HSK21542 dose groups experienced 85 TEAEs, with TEAEs occurring in 83.3%, 75.0%, 83.3% and 66.7% of the patients in HSK21542 dosage groups 1∼4, respectively, while 9 (75.0%) patients in the placebo group experienced 31 TEAEs (Table 2). The severity of TEAEs were mostly grade 1 or 2, with grade 3 TEAEs reported only in a small number of patients in HSK21542-group 1 (25.0%), HSK21542-group 2 (8.3%) and the placebo group (16.7%). The most common TEAEs were fever (22.9% vs. 41.7%), nausea (25.0% vs. 33.3%) and vomiting (22.9% vs. 25.0%) in the HSK21542 and placebo groups. The incidence of drug-related TEAEs was 12.5% in the HSK21542 dose groups, occurring in 16.7%, 16.7%, 8.3% and 8.3% of patients in HSK21542 groups 1∼4, respectively, while the incidence was 16.7% in the placebo group. The incidence of drug-related nausea and vomiting were 2.1% vs. 8.3% and 8.3% vs. 8.3% in the HSK21542 and placebo groups, respectively (Supplementary Table 1). None of the patients experienced ≥ grade 3 drug-related TEAEs, and most of the TEAEs recovered without treatment or after short-term treatment without obvious clinical symptoms and signs. For SS in stage 1, the proportion of anti-emetic drugs administered to the HSK21542 dose groups (25.0%) was lower than in the placebo (41.7%) within 0–24 h after the first postoperative dosing of experimental drugs (Table 2). Correspondingly, the mean (± SD) cumulative dosage of tropisetron in the HSK21542 dose groups was lower than in the placebo (1.7 ± 2.5 mg) within 0–24 h after the first postoperative dosing of experimental drugs, and especially lower in the HSK21542-group 3 (0.4 ± 1.0 mg) and HSK21542-group 4 (0.4 ± 1.0 mg) (Supplementary Figure 1).

**TABLE 2:** Summary of treatment-emergent adverse events of enrolled patients in stage 1 and stage 2 (safety set).

Variables	Stage 1						Stage 2		
	Placebo group (*n* = 12)	HSK21542 group					Placebo group (*n* = 20)	HSK21542-0.5 μg/kg group (*n* = 20)	HSK21542-1.0 μg/kg group (*n* = 20)
		Group 1 (*n* = 12)	Group 2 (*n* = 12)	Group 3 (*n* = 12)	Group 4 (*n* = 12)	Total (*n* = 48)			
Any AEs, *n* (%)	10 (83.3)	11 (91.7)	9 (75.0)	11 (91.7)	8 (66.7)	39 (81.3)	18 (90.0)	17 (85.0)	16 (80.0)
Any TEAEs, *n* (%)	9 (75.0)	10 (83.3)	9 (75.0)	10 (83.3)	8 (66.7)	37 (77.1)	16 (80.0)	17 (85.0)	15 (75.0)
Grade 1	8 (66.7)	7 (58.3)	8 (66.7)	7 (58.3)	5 (41.7)	27 (56.3)	15 (75.0)	12 (60.0)	14 (70.0)
Grade 2	7 (58.3)	7 (58.3)	4 (33.3)	6 (50.0)	5 (41.7)	22 (45.8)	9 (45.0)	11 (55.0)	5 (25.0)
Grade 3	2 (16.7)	3 (25.0)	1 (8.3)	0	0	4 (8.3)	2 (10.0)	0	0
Drug-related TEAEs, *n* (%)	2 (16.7)	2 (16.7)	2 (16.7)	1 (8.3)	1 (8.3)	6 (12.5)	2 (10.0)	0	3 (15.0)
Grade 1	2 (16.7)	0	2 (16.7)	1 (8.3)	0	3 (6.3)	1 (5.0)	0	3 (15.0)
Grade 2	1 (8.3)	2 (16.7)	1 (8.3)	0	1 (8.3)	4 (8.3)	1 (5.0)	0	1 (5.0)
Grade 3	0	0	0	0	0	0	0	0	0
Any SAEs	1 (8.3)	0	0	0	0		1 (5.0)	0	0
TEAEs leading to treatment interruptions, *n* (%)	0	0	0	0	0	0	0	0	0
TEAEs resulting in study discontinuation, *n* (%)	0	1 (8.3)	0	0	0	1 (2.1)	0	0	0
Antiemetic used, *n* (%)									
0–12 h after first postoperative dosing of experimental drugs	5 (41.7)	5 (41.7)	3 (25.0)	2 (16.7)	1 (8.3)	11 (22.9)	6 (30.0)	5 (25.0)	2 (10.0)
0–24 h after first postoperative dosing of experimental drugs	5 (41.7)	5 (41.7)	3 (25.0)	2 (16.7)	2 (16.7)	12 (25.0)	6 (30.0)	5 (25.0)	3 (15.0)
**TEAEs occurring in > 10% of patients, termed by PT, *n* (%)**									
Fever	5 (41.7)	1 (8.3)	4 (33.3)	3 (25.0)	3 (25.0)	11 (22.9)	3 (15.0)	1 (5.0)	4 (20.0)
Nausea	4 (33.3)	3 (25.0)	4 (33.3)	2 (16.7)	3 (25.0)	12 (25.0)	4 (20.0)	2 (10.0)	4 (20.0)
Vomiting	3 (25.0)	5 (41.7)	3 (25.0)	1 (8.3)	2 (16.7)	11 (22.9)	8 (40.0)	6 (30.0)	4 (20.0)
Decreased free triiodothyronine	2 (16.7)	1 (8.3)	0	2 (16.7)	2 (16.7)	5 (10.4)	1 (5.0)	1 (5.0)	2 (10.0)
Decreased serum potassium	1 (8.3)	2 (16.7)	0	1 (8.3)	2 (16.7)	5 (10.4)	3 (15.0)	0	1 (5.0)
Elevated WBC counts	1 (8.3)	2 (16.7)	0	1 (8.3)	0	3 (6.3)	1 (5.0)	1 (5.0)	2 (10.0)
Decreased heart rate	1 (8.3)	2 (16.7)	0	0	0	2 (4.2)	0	1 (5.0)	0
Anemia	0	0	0	1 (8.3)	1 (8.3)	2 (4.2)	3 (15.0)	4 (20.0)	1 (5.0)

For HSK21542 dose groups in stage 1: group 1: preoperative 0.4 μg/kg + 0.2 μg/kg at postoperative 0 h, 8 h and 16 h; group 2: preoperative 1.0 μg/kg + 0.5 μg/kg at postoperative 0 h, 8 h and 16 h; group 3: 0.5 μg/kg at postoperative 0 h, 8 h and 16 h; group 4: 1.0 μg/kg at postoperative 0 h, 8 h and 16 h. Stage 2: HSK21542-0.5 μg/kg or HSK21542-1.0 μg/kg were administered postoperatively at 0 h, 8 h and 16 h. AE, adverse event; SAE, serious adverse event; TEAE, treatment-emergent adverse event.

Among the SS in stage 2, the incidences of TEAEs were 85.0%, 75.0% and 80.0%, and the corresponding drug-related TEAEs occurrences were 0%, 15.0% and 10.0% in the HSK21542-0.5 μg/kg, HSK21542-1.0 μg/kg and placebo groups, respectively (Table 2). The majority of TEAEs were self-limiting and only 2 (10.0%) patients in the placebo group experienced ≥ grade3 TEAEs. The most common TEAEs were vomiting (30.0% vs. 20.0% vs. 40.0%), nausea (10.0% vs. 20.0% vs. 20.0%), fever (5.0% vs. 20.0% vs. 15.0%) and anemia (20.0% vs. 5.0% vs. 15.0%) in the HSK21542-0.5 μg/kg, HSK21542-1.0 μg/kg and placebo groups, respectively; it should be noted that none of these TEAEs were related to the experimental drugs (placebo or HSK21542). The proportion of anti-emetic drugs given was lowest in the HSK21542-1.0 μg/kg group (15.0%), followed by the HSK21542-0.5 μg/kg (25.0%) and placebo groups (30.0%) within 0–24 h after the first postoperative dosing of experimental drugs (Table 2). Correspondingly, the mean (± SD) cumulative dosage of tropisetron in the HSK21542 dose groups was lower than in the placebo group (1.1 ± 2.1 mg) within 0–24 h after the first postoperative dosing of experimental drugs, with the lowest values occurring in the HSK21542-1.0 μg/kg group (0.4 ± 0.9 mg) (Supplementary Figure 1).

There were no deaths in either stage 1 or stage 2, and serious AEs (SAEs) only occurred in the placebo group, with one case of papillary thyroid carcinoma (related to the primary disease) in stage 1 and 1 case of post-operative infection in stage 2. In this study, TEAEs related to sedation, anxiety and diuresis were rarely reported in stages 1 and 2. Only 1 case of frequent urination (grade 2) occurred in the HSK21542-group 4 in stage 1, and although this case was related to the HSK21542 administration, the outcome was recovery after continuation of administration of HSK21542 according to the dosing schedule.

All clinically significant abnormal values from laboratory, vital signs and ECG values were reported as TEAEs. The changes in laboratory tests from baseline were also analyzed, and no significant differences were observed between the HSK21542 and placebo groups in stage 1 and stage 2. Vital signs were stable in all the patients throughout stages 1 and 2, no dose-dependent effects on vital signs and laboratory parameters were observed, and no clinically significant QTcF interval prolongation or physical examination anomalies were noted.

### 3.3 Efficacy

After pooling the data from stage 1 and stage 2 for patients who received HSK21542-0.5 μg/kg and HSK21542-1.0 μg/kg postoperatively, it was found that the mean (± SD) values of SPID_0–24__*h*_ in the HSK21542-1.0 μg/kg group (−1,679.8 ± 2,284.3 scores × min) were apparently lower than in the placebo (−435.2 ± 2,852.9 scores × min) and HSK21542-0.5 μg/kg groups (−1,499.4 ± 2,487.2 scores × min), but a statistically significant difference was not achieved (*P* = 0.114). It is worth noting that PID at most times points in the HSK21542-1.0 μg/kg group were lower than in the HSK21542-0.5 μg/kg and placebo groups, which especially had a significantly higher LSMD of PID when compared to the placebo group (0.61; *P* = 0.020) (Table 3).

**TABLE 3:** Efficacy outcomes of patients pooled from stage 1 and stage 2 (FAS).

	Placebo (*n* = 32)	HSK21542-0.5 μg/kg group (*n* = 32)	HSK21542-1.0 μg/kg group (*n* = 32)	*P*-value
SPID_0–24__*h*_ (scores × min)				0.101
Mean (SD)	−435.2 (2,852.9)	−1,499.4 (2,487.2)	−1,679.8 (2,284.3)	
Median (range)	−941.5 (−8,926–3,903)	−1,417.0 (−7,472–3,939)	−1,416.0 (−6,311–3,971)	
SPID_0–12__*h*_ (scores × min)				0.114
Mean (SD)	−29.4 (1,455.3)	−530.2 (1,118.5)	−682.8 (1,185.6)	
Median (range)	12.5 (−4,556–2,628)	−504.5 (−2,826–2,091)	−675.0 (−2,824–2,234)	
**PID (scores × min)**				
LSMD vs. placebo (95% CI)		0.44 (−0.05, 0.92)	0.61 (0.10, 1.12)	
*P* value vs. placebo*		0.080	0.020	
Patients administered with morphine 0–12 h after first dosing of the experimental drugs, *n* (%)	14 (43.8)	10 (31.3)	6 (18.8)	0.100
Cumulative dosage of morphine 0–12 h after first dosing of the experimental drugs (mg)				0.111
Mean (SD)	1.9 (2.9)	1.2 (2.0)	0.8 (1.9)	
Median (range)	0.0 (0–11)	0.0 (0–8)	0.0 (0–8)	
Patients administered with morphine 0–24 h after first dosing of experimental drugs, *n* (%)	14 (43.9)	11 (34.4)	6 (18.8)	0.097
Cumulative dose of morphine 0–24 h after first postoperative dosing of the experimental drugs (mg)				
Mean (SD)	2.5 (3.7)	1.4 (2.2)	1.0 (2.6)	0.085
Median (range)	0.0 (0–14)	0.0 (0–8)	0.0 (0–12)	
Time of first injection of morphine (min)				0.654
Mean (SD)	129.0 (145.6)	216.1 (337.3)	217.2 (205.1)	
Median (range)	75.9 (1.9–509.8)	86.8 (4.1–1179.9)	195.6 (15.3–588.2)	

*Data were compared using mixed model repeated measures (MMRM) methods, with the baseline pain score, treatment, time point and interaction between treatment and time point as fixed effects, and patients as random effects. Statistical significance was defined as a two-sided alpha level of *P* < 0.05. AUC, area under curve; LSMD, least squares mean difference; NRS, numerical rating scale; PID, pain intensity difference; SD, standard deviation; SPID_0–12__*h*_, time-weighted summed pain intensity differences over 12 h; SPID_0–24__*h*_, time-weighted summed pain intensity differences over 24 h.

**FIGURE 2:**
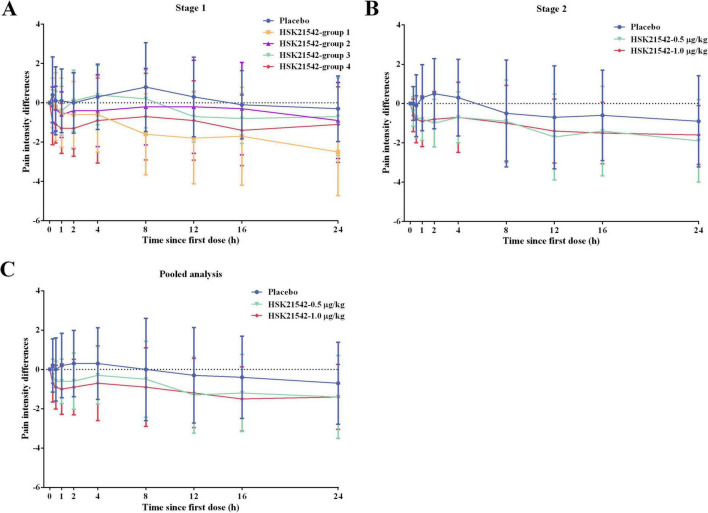
The PID curves over time for enrolled patients in **(A)** stage 1, **(B)** stage 2, and **(C)** pooled data from stages 1 and 2. For HSK21542 dose groups in stage 1: group 1: preoperative 0.4 μg/kg + 0.2 μg/kg at postoperative 0 h, 8 h and 16 h; group 2: preoperative 1.0 μg/kg + 0.5 μg/kg at postoperative 0 h, 8 h and 16 h; group 3: 0.5 μg/kg at postoperative 0 h, 8 h and 16 h; group 4: 1.0 μg/kg at postoperative 0 h, 8 h and 16 h. Stage 2: HSK21542-0.5 μg/kg or HSK21542-1.0 μg/kg were administered postoperatively at 0 h, 8 h and 16 h.

For FAS in stage 1, mean values of SPID_0–12__*h*_ and SPID_0–24__*h*_ in the all HSK21542 dose groups were lower than those in the placebo group, which also appeared to have a slightly longer duration of analgesia, higher patient and physician satisfaction scores than the placebo group, but the apparent differences were not statistically significant (Supplementary Table 2). The mean (± SD) values of SPID_0–12__*h*_ and SPID_0–24__*h*_ in HSK21542-group 4 (−621.3 ± 1,303.7 and −1,394.8 ± 2,536.3 scores × min) were lower than HSK21542-group 2 (−177.6 ± 1,394.4 and −695.1 ± 2,749.8 scores × min) and HSK21542-group 3 (−95.3 ± 866.7 and −620.8 ± 1,948.1 scores × min). Moreover, PID at all time points in HSK21542-group 4 were lower than groups 2 and 3, and MMRM analysis also revealed the highest least squares mean difference (LSMD) of PID results in HSK21542-group 4 (0.55; *P* = 0.272) compared to the placebo (groups 1–3: 0.34 vs. 0.37 vs. 0.45) (Figure 2A and Supplementary Table 2). The proportion of patients in HSK21542-group 4 who had an NRS ≤ 3 (66.7%) during 0–24 h postoperative dosing of HSK21542 was higher than that in HSK21542-group 2 (58.3%) and HSK21542-group 3 (58.3%). The median time to first use of morphine for rescue analgesia in the HSK21542-group 4 was longer than for all the other groups. Correspondingly, the proportion of morphine used for rescue analgesia and the mean cumulative dosage in the HSK21542-group 4 were both lowest (Supplementary Table 2).

For FAS in stage 1, HSK21542-group 1 had the lowest mean (± SD) values of SPID_0–12__*h*_ (−972.5 ± 1,334.7 scores × min) and SPID_0–24__*h*_ (−2,487.8 ± 2,930.0 scores × min), while the mean cumulative dosage of morphine, the proportion of patients receiving morphine, and the proportion of patients with an NRS score ≤ 3 at 0–24 h after the first postoperative dosing of experimental drugs were higher than those in the placebo group and the other HSK21542 dose groups. Of note, the median time to first use of morphine for rescue analgesia was only 15.5 min in the HSK21542-group 1 (Supplementary Table 2).

For FAS in stage 2, the efficacy outcomes in the two HSK21542 groups were superior to the placebo group. The mean (± SD) values of SPID_0–12__*h*_ and SPID_0–24__*h*_ in HSK21542-0.5 μg/kg (−791.1 ± 1189.7 and −2,026.6 ± 2,667.5 scores × min) and HSK21542-1.0 μg/kg (−719.8 ± 1,142.6 and −1,850.8 ± 2,169.6 scores × min) groups were lower than those in the placebo group (−223.3 ± 1,544.0 and −772.9 ± 3,108.1 scores × min), but the apparent difference did not reach statistical significance (all *P* > 0.05; Table 4). The PID of the HSK21542-1.0 μg/kg group was similar to that of the HSK21542-0.5 μg/kg group at most time points, which were both lower than the placebo group at all time points (Figure 2). MMRM analysis also demonstrated that patients in the HSK21542-1.0 μg/kg group had the highest LSMD of PID results (0.59 vs. 0.45) compared to the placebo group (*P* = 0.061). In addition, compared to the HSK21542-0.5 μg/kg group, the HSK21542-1.0 μg/kg group had a longer median time to first morphine rescue, a slightly lower proportion of patients using morphine within 0–24 h after the first dosing of experimental drugs, as well as a slightly lower cumulative dosage of morphine (Table 4). Moreover, in patients in the HSK21542-1.0 μg/kg group had a slightly longer duration of analgesia, higher proportion of patients with NRS ≤ 3, and higher patient and physician satisfaction scores than those of the HSK21542-0.5 μg/kg group at 0–24 h after the first postoperative administration of the experimental drugs.

**TABLE 4:** Efficacy outcomes of patients enrolled in stage 2 (FAS).

	Placebo (*n* = 20)	HSK21542-0.5 μg/kg group (*n* = 20)	HSK21542-1.0 μg/kg group (*n* = 20)	*P*-value
SPID_0–24__*h*_ (scores × min)				0.406
Mean (SD)	−772.9 (3,108.1)	−2,026.6 (2,667.5)	−1,850.8 (2,169.6)	
Median (range)	−1,192.0 (−8,926–3,606)	−1,502.0 (−7,472–3,939)	−1,660.0 (−6,311–2,168)	
SPID_0–12__*h*_ (scores × min)				0.518
Mean (SD)	−223.3 (1,544.0)	−791.1 (1,189.7)	−719.8 (1,142.6)	
Median (range)	−258.5 (−4,556–1902)	−716.5 (−2,826–2,091)	−675.0 (−2,824–1,922)	
**PID (scores × min)**				
LSMD vs. placebo (95% CI)		0.45 (−0.21, 1.10)	0.59 (−0.03, 1.20)	
*P*-value vs. placebo*		0.175	0.061	
Patients administered with morphine 0–12 h after first dosing of experimental drugs, *n* (%)	10 (50.0)	6 (30.0)	4 (20.0)	0.123
Cumulative dosage of morphine 0–12 h after first dosing of the experimental drugs (mg)				0.115
Mean (SD)	2.5 (3.4)	1.3 (2.3)	0.9 (2.0)	
Median (range)	1.5 (0–11)	0.0 (0–8)	0.0 (0–8)	
Patients administered with morphine during 0–24 h after first dosing of experimental drugs, *n* (%)	10 (50.0)	6 (30.0)	4 (20.0)	0.123
Cumulative dose of morphine 0–24 h after first dosing of the experimental drugs (mg)				0.109
Mean (SD)	3.0 (4.2)	1.5 (2.5)	0.9 (2.0)	
Median (range)	1.5 (0–11)	0.0 (0–8)	0.0 (0–8)	
Time of first injection of morphine (min)				0.883
Mean (SD)	78.2 (81.9)	103.9 (134.5)	133.6 (120.3)	
Median (range)	59.6 (1.9–286.3)	61.7 (4.1–362.5)	129.1 (15.3–260.9)	
Patients with NRS ≤ 3 during 0–12 h administration, *n* (%)	10 (50.0)	12 (60.0)	14 (70.0)	0.435
Patients with NRS ≤ 3 during 0–24 h administration, *n* (%)	10 (50.0)	12 (60.0)	14 (70.0)	0.435
Analgesia duration (min)				0.210
Mean (SD)	1,306.2 (234.6)	1,324.8 (173.0)	1,406.2 (68.1)	
Median (range)	1,420.2 (623.0–1,463.8)	1,409.9 (836.0–1,442.4)	1,422.9 (1,212.0–1,496.0)	
Satisfaction score for patients				0.377
Mean (SD)	9.1 (1.3)	8.7 (1.8)	9.4 (1.1)	
Median (range)	9.5 (6–10)	9.0 (5–10)	10.0 (6–10)	
Satisfaction score for clinician				0.290
Mean (SD)	8.6 (2.0)	8.3 (1.7)	9.1 (1.5)	
Median (range)	10.0 (4–10)	9.0 (5–10)	10.0 (6–10)	

*Data were compared using mixed model repeated measures (MMRM) methods, with the baseline pain score, treatment, time point and interaction between treatment and time point as fixed effects, and patients as random effects. Statistical significance was defined as a two-sided alpha level of *P* < 0.05. For HSK21542 dose groups in stage 2: HSK21542-0.5 μg/kg or HSK21542-1.0 μg/kg were administered at postoperative 0 h, 8 h and 16 h. AUC, area under curve; LSMD, least squares mean difference; NRS, numerical rating scale; PID, pain intensity difference; SD, standard deviation; SPID_0–12__*h*_, time-weighted summed pain intensity differences over 12 h; SPID_0–24__*h*_, time-weighted summed pain intensity differences over 24 h.

### 3.4 Plasma concentrations of HSK21542 and prolactin level

Except for the HSK21542-group 1, the plasma concentration of HSK21542 in the HSK21542 dose groups increased sharply immediately after the third postoperative dose of HSK21542 and decreased rapidly 24 h after the first postoperative dose of HSK21542 in stage 1 and stage 2 (Supplementary Figure 2). Patients in HSK21542-group 1 did not have a decrease in plasma concentrations of HSK21542 from the time of the first dose to the time of the third postoperative dose of HSK21542, and did not exhibit an increase immediately following the third dose after surgery. In stage 2, it was found that the plasma concentration of HSK21542-1.0 μg/kg group was nearly twice that of the HSK21542-0.5 μg/kg group immediately after the third postoperative dose of HSK21542, which was not observed in stage 1.

There was no significant dose correlation in changes in serum prolactin concentrations after treatment with HSK21542 in stage 1, but notably patients in the HSK21542 dose groups exhibited greater elevations in prolactin concentrations from baseline than patients in the placebo group (Supplementary Figure 3).

## 4 Discussion

The present placebo-controlled, two-stage phase 2 clinical trial demonstrated that HSK21542 was well tolerated with a low incidence of postoperative nausea and vomiting (PONV) and good postoperative analgesia efficacy (24 h-period). HSK21542-1.0 μg/kg administered at postoperative periods 0 h, 8 h and 16 h was superior to placebo in patients undergoing elective laparoscopic abdominal surgery.

The most concerning complications after laparoscopic abdominal surgery were PONV; furthermore, opioids may increase the risk of PONV (17). In the present trial, patients in the HSK21542 groups enrolled from stage 1 and stage 2 exhibited a lower incidence of nausea and/or vomiting, which were in close agreement with findings for the other selective peripherally-restricted KOR agonist (18, 19). In addition, the incidence of PONV with HSK21542 was not dose-dependent in stage 1 and was lower in patients receiving postoperative administration (groups 3 and 4) compared to those patients receiving both preoperative and postoperative administration (groups 1 and 2). Significantly, the cumulative dosage of morphine used for rescue analgesia was higher in the HSK21542 group 1 (4.3 mg) and group 2 (1.6 mg), and was even higher in group 1 than in the placebo group (1.8 mg), which may explain the higher incidence of nausea and/or vomiting. Since previous studies have shown that μ-opioid receptor (MOR) agonists (such as morphine) could either stimulate MOR in the gastrointestinal tract to inhibit gastrointestinal motility or activate MOR in the chemoreceptor trigger zone, thereby leading to a relative higher incidence of PONV (20–22). The present trial also demonstrated that the cumulative doses of anti-emetic drugs in all HSK21542 groups was lower than those in placebo group within 0–24 h after the first postoperative administration of experimental drugs, with the lowest values especially observed in the HSK21542-1.0 μg/kg group. Up to now, whether KOR agonists decrease the risk of PONV is uncertain, and one hypothesis is that activation of KOR could act directly on the vagus nerve, thereby altering the CNS regulation of gastrointestinal stimulatory signals and ameliorating early intestinal dilatation and flatulence caused by gastrointestinal discomfort after abdominal surgery or anesthesia (23). In addition to the above findings, the majority of AEs were grade 1 or grade 2 in severity, and most TEAEs were alleviated without treatment or after short-term treatment, without obvious clinical symptoms and signs. Thus, HSK21542 administered postoperatively was well tolerated, with a lower incidence of PONV in patients undergoing laparoscopic abdominal surgery.

During stage 1, it was found that the mean (± SD) values of SPID_0–12 *h*_ were lower in the HSK21542-group 2 (−177.6 ± 1,394.4 vs. −95.3 ± 866.7 scores × min), but the SPID_0–24 *h*_ was comparable between HSK21542-group 2 and group 3 (−695.1 ± 2,749.8 vs. −620.8 ± 1,948.1 scores × min). However, the LSMD of PID results when compared to placebo was slightly higher in the HSK21542-group 3 (0.45 vs. 0.37), and accumulative dosages of morphine used for rescue analgesia (1.3 vs. 1.6 mg) in the HSK21542-group 3 were also lower than for the HSK21542-group 2. All the above results indicated that increasing the frequency of preoperative administration of HSK21542 did not improve the efficacy of postoperative analgesia. Of note, patients in HSK21542-group 1 had the lowest values of SPID_0–12 *h*_ and SPID_0–24 *h*_, but it also led to excessive cumulative dosage of morphine rescue and related PONV, which may be related to the accumulation of plasma concentration of HSK21542 and individual heterogeneity in group 1, a conjecture that warrants further investigation. To sum up, HSK21542 doses of 0.5 μg/kg and 1.0 μg/kg are recommended to be administered postoperatively at 0, 8 and 16 h for the subsequent stage 2 trial. The data from stage 1 and stage 2 were pooled and it was found that statistically significant PID results were observed in the HSK21542-1.0 μg/kg group compared to the placebo group (LSMD: 0.61, 95% CI: 0.10, 1.12, *P* = 0.020), and were accompanied by the lowest dosage of morphine rescue and a slightly higher satisfactory rating. A linear PK characteristic was also found in the dose-dependent trend of plasma concentration between the HSK21542-0.5 μg/kg and HSK21542-1.0 μg/kg groups. Therefore, HSK21542-1.0 μg/kg administered postoperatively at 0, 8 and 16 h was recommended for the phase 3 trial, with a larger sample size to verify and strengthen statistical significance.

There were several limitations to the present research. The phase 2 trial was an exploratory study with a smaller sample size, and the calculation of sample size was not based on the statistical power. The study design initially required inclusion of patients undergoing laparoscopic abdominal surgery, and the inclusion criteria did not limit the types of laparoscopic abdominal surgery, but ultimately the actual enrolled patients where all female underwent laparoscopic hysterectomy, even some enrolled patients also underwent laparoscopic salpingectomy, pelvic lymphadenectomy, lysis of pelvic adhesions, and other laparoscopic gynecologic surgery. However, considering that laparoscopic surgery is a common surgical procedure in gynecology department, gynecological surgery is also the recommended pain model for mixed somatic and visceral pain in the guidelines for analgesic drug development in China and European. Therefore, although the type of surgery included in the study is a representative laparoscopic abdominal surgery and a representative pain model for analgesia studies, it limits the generalizability of the study population, warrants further investigations. It is well known that females have an increased sensitivity to pain perception than males, as well as a higher incidence of acute postoperative pain (24). However, the research findings regarding the gender difference in the analgesic response of opioid drugs were inconclusive, and mixed κ-opioid agonist-antagonist such as buprenorphine, nalbuphine and pentazocine produced better analgesic effects for females (25, 26). Therefore, the generalizability of the findings should be interpreted with caution. In future studies, a stratified randomization approach will be adopted, with gender and the types of laparoscopic surgery as stratification factors, to determine the equilibrium of the characteristics of enrolled patients in different groups. In addition, the present trial assessed safety and efficacy in the short postoperative period (8 ± 1 days) without the development of chronic postoperative pain, which requires further confirmation with longer follow-ups and a larger cohort size involving more surgical types.

In conclusion, HSK21542 at all dose regimens was well tolerated with a lower incidence of PONV than placebo, as well as a requirement for a reduced dosage of relative anti-emetic medication in the phase 2 trial. In particular, patients who received HSK21542-1.0 μg/kg at 0, 8 and 16 h after surgery had more effective analgesia following laparoscopic abdominal surgery, with a relatively smaller dosage of morphine needed for rescue, and also fewer anti-emetic drugs. It should be noted that although HSK21542-1.0 μg/kg showed better analgesic effect, this apparent difference did not reach statistical significance, but a more effective dose and postoperative administration mode of HSK21542 were initially determined in this exploratory study, which can be used as a reference for further large-scale studies. Therefore, the dosing regimen of HSK21542-1.0 μg/kg administered postoperatively at 0 h, 8 h and 16 h exhibited an acceptable efficacy, warranting its recommendation for further phase 3 trial with larger, more diverse populations.

## Data Availability

The original contributions presented in the study are included in the article/Supplementary material, further inquiries can be directed to the corresponding author.
