# Supplementary material for: Efficacy and safety of peripherally-restricted κ-opioid receptor agonist-HSK21542 for postoperative analgesia in patients undergoing laparoscopic abdominal surgery: a randomized, placebo-controlled phase 2 trial
Source: Front Med (Lausanne). 2025 Jul 24;12:1604790. doi: 10.3389/fmed.2025.1604790 (PMC12328421; doi:10.3389/fmed.2025.1604790)
Supplement: Supplementary file 1 [file Data_Sheet_1.pdf]

Efficacy and safety of peripherally-restricted κ-opioid receptor agonist-HSK21542 for postoperative analgesia in patients undergoing laparoscopic abdominal surgery: a randomized, placebo-controlled phase 2 trial

Running title: HSK21542 for postoperative analgesia

Yinbo Zhong[1†], Haiying Wang[1†], Min Yan[1*], Mengchang Yang[2], Jiaqiang Zhang[3], Ling Nan[4], Zhiping Wang[5], Jianjun Yang[6], Jinglei Wu[7], Qulian Guo[8], Xiaoling Hu[9], Hongmeng Xu[10], Qiang Xu[11], Dongxin Wang[12]

[1]Department of Anesthesiology, The Second Affiliated Hospital, Zhejiang University School of Medicine, Hangzhou, China
[2]Department of Anesthesiology, Sichuan Provincial People's Hospital, School of Medicine, University of Electronic Science and Technology of China, Chengdu, China
[3]Department of Anesthesiology, Pain and Perioperative Medicine, Henan Provincial People's Hospital, Zhengzhou, China
[4]Department of Anesthesiology, The First Bethune Hospital of Jilin University, Changchun, China
[5]Department of Anesthesiology, The Affiliated Hospital of Xuzhou Medical University, Xuzhou, China
[6]Department of Anesthesiology, Pain and Perioperative Medicine, The First Affiliated Hospital of Zhengzhou University, Zhengzhou, China
[7]Department of Anesthesiology, Liuzhou People's Hospital Affiliated to Guangxi Medical University, Liuzhou, China
[8]Department of Anesthesiology, Xiangya Hospital, Central South University, Changsha, China
[9]Department of Anesthesiology, The First Affiliated Hospital of University of South China, Hengyang, China
[10]Department of Anesthesiology, The Fourth Hospital of Hebei Medical University, Shijiazhuang, China
[11]Department of Anesthesiology, Union Hospital, Tongji Medical College, Huazhong University of Science and Technology, Wuhan, China
[12]Department of Anesthesiology, Peking University First Hospital, Beijing, China

[†]These authors contributed equally to this work and share first authorship
[*]Correspondence:
Prof. Min Yan, Ph.D.
Email: zryanmin@zju.edu.cn

Supplementary Appendix 1. Inclusion and exclusion criteria

1. Inclusion criteria. Patients who met all of the following criteria were included:

1) Patients with an age of ≥ 18 or ≤ 70 years, with no gender requirement;
2) American Society of Anesthesiologists (ASA) Class I-II;
3) 18 kg/m^2 ≤ BMI ≤ 40 kg/m^2;
4) Patients who were due to undergo elective laparoscopic abdominal surgery under general anesthesia, with an expected surgery duration of 1-5 h (inclusive);
5) Agreed to participate in the trial and voluntarily signed an informed consent form;

2. Exclusion criteria. Patients who met any of the following criteria were excluded from the trial:

1) Had a history of allergy to opioids, such as urticaria, or were allergic to the intraoperative anesthetics described in the protocol;
2) History or evidence of any one of the following diseases prior to screening:
   a) History of cardiovascular diseases: uncontrolled hypertension (systolic blood pressure [SBP] ≥ 170 mmHg and/or diastolic blood pressure [DBP] ≥ 105 without antihypertensive treatment, or SBP > 160 mmHg and/or DBP > 100 mmHg despite antihypertensive treatment); aneurysm; severe arrhythmia; heart failure; Adams-Stokes syndrome, New York Heart Association (NYHA) Class ≥ III; severe superior vena cava syndrome; pericardial effusion; acute myocardial ischemia; unstable angina; myocardial infarction in the last 6 months before screening or a history of tachycardia/bradycardia requiring medication; and degree II-III atrioventricular block (excluding patients with pacemakers);
   b) Had a history of respiratory disorders: severe chronic obstructive pulmonary disease; acute exacerbation of chronic obstructive pulmonary disease; severe airway constriction; throat mass; history of (bronchial) tracheoesophageal fistula or airway tear; or severe respiratory tract infection in the last 2 weeks before screening;
   c) Had a history of disorders of the nervous systems of psychiatric problems: history of craniocerebral injury; possible convulsions; intracranial hypertension; cerebral aneurysms and a history of cerebrovascular accidents; history of schizophrenia; mania; mental aberrations; long-term use of psychotropic drugs; cognitive disorders; history of depression, anxiety and epilepsy, etc.;
   d) Patients who had undergone major surgery within 3 months before screening that was judged by the investigator to affect the assessment of postoperative pain;
3) Any of the following airway management risks during screening:
   a) Acute exacerbation of asthma;
   b) Sleep apnea syndrome;
   c) Had a history or family history of malignant high fever;
   d) Patients with a failed tracheal intubation experience;
   e) Difficult airway as judged by the investigator (e.g., Modified Mallampati Score ≥ III);
4) Any of the following drugs or treatments were used during the screening period:
   a) The time from the last use of an opioid or non-opioid (e.g., such as acetaminophen, aspirin [daily dose of > 100 mg], indomethacin, diclofenac, parecoxib sodium and other non-steroidal anti-inflammatory drugs) analgesic

drug was shorter than 5 half-life periods or the duration of the drug effect (whichever was the longest);

b) Use of opioid analgesics for any reason for > 10 consecutive days within 3 months before screening;

c) The use of drugs that may affect analgesia with an unclear half-life within 14 days before randomization, or the use of drugs that affect analgesia before randomization, and the last use time was < 5 half-lives from randomization (according to the drug inserts and reference materials, irrational drug use was analysed.), including but not limited to: sedative hypnotics (benzodiazepines [triazolam, diazepam, midazolam, etc.]; non-benzodiazepines [zolpidem, zopiclone, zaleplon, etc.]); sedative anesthetics (sevoflurane, anesthesia ether, nitrous oxide, thiopental, ketamine, etomidate, etc.); glucocorticoids (dexamethasone hydrochloride, methylprednisolone, etc.); anti-epilepsy drugs (carbamazepine, sodium valproate, etc.); anxiolytics (carbamazepine, diazepam, etc.); antidepressants (imipramine, amitriptyline, etc.); and Chinese herbal medicines or Chinese patent medicines with analgesic and sedative effects;

d) Drugs and treatments with antitumor effects, including but not limited to chemotherapeutic agents, targeted agents, and Chinese herbal medicines, were expected to be required from 14 days before randomization to the end of the follow-up period;

e) The time between randomization and last use of a diuretic or diuretic containing combination drug was < five half-lives of the drug or the duration of the drug effect, whichever was the longest;

5) During the screening period, the laboratory test indicators met one of the following criteria, confirmed by reexamination:
   a) White blood cell count < 3.0×10^9/L;
   b) Platelet count < 80×10^9/L;
   c) Hemoglobin < 70 g/L;
   d) Prothrombin time > 1.5 × upper limit of normal (ULN);
   e) Activated partial thromboplastin time > 1.5 × ULN;
   f) Alanine aminotransferase and/or aspartate aminotransferase > 2 × ULN;
   g) Total bilirubin > 1.5 × ULN;
   h) Serum creatinine > 1.5 × ULN;
   i) Fasting blood glucose ≥ 11.1 mmol/L;
6) During the screening period, the pulse oxygen saturation was < 92% without supplemental oxygen;
7) Hepatitis C antibody (HCVAb), syphilis antibody and human immunodeficiency virus (HIV) antibody were positive during the screening period;
8) Had a history of drug abuse, drug addiction, and/or alcohol abuse, which was defined as drinking an average of > 2 units of alcohol per day (1 unit = 360 mL of 5% beer or 45 mL of 40% liquor or 150 mL of wine) in the 3 months prior to the screening period;
9) Had a history of blood donation or blood loss ≥ 400 mL within 3 months before screening;
10) Had participated in any pharmaceutical clinical trial (defined as receipt of a trial drug or placebo) within 3 months before screening;

11) Pregnant and lactating women; women or men of childbearing potential who were unwilling to use contraception throughout the study period; or planned pregnancy within 3 months of the end of the study (including males);
12) Patients with any other factors considered by the investigator to be ineligible for participation in the clinical trial.

Supplementary Appendix 2. The assessment methods of adverse events and other safety indicators, plasma concentration of HSK21542 and prolactin concentrations

Laboratory test included the blood routine, urine routine, blood biochemistry, blood electrolytes, thyroid function and coagulation function. Laboratory test was carried out during screening and treatment periods (Day 3). Blood electrolytes were evaluated within 1 h before induction of anesthesia, within 30 min before the last postoperative dose, 24 h (± 1 h) and 3 days after the first postoperative dose of HSK21542. Thyroid function was evaluated within 1 h before induction of anesthesia, 24 h (± 1 h) and 3 days after the first postoperative dose of HSK21542. 12-ECG was evaluated at screening period, within 1 h before induction of anesthesia, within 15 min before the last postoperative dose, 24 h (± 1 h) and 3 days after the first postoperative dose of HSK21542. Vital signs were evaluated within 1 h before induction of anesthesia, 0 min (within 10 min before the first postoperative dose), 8 h (within 30 min before the second postoperative dose), 16 h (within 30 min before the last postoperative dose), 24 h (± 1 h) and 3 days after the first postoperative dose of HSK21542. The plasma concentration of HSK21542 was detected using methodological validated liquid chromatography-tandem mass spectrometry (LC-MS/MS), with a low limit of quantification (LLOQ) of 0.05 ng/mL. In stage 1, blood samples were collected before induction of anesthesia, immediately after the first dose of HSK21542 (groups 1 and 2 were administered preoperatively and groups 3 and 4 were administered postoperatively), before and immediately after the third postoperative dose of HSK21542, and 24 h after the first postoperative dose of HSK21542 for the analysis of the plasma concentration of HSK21542, and prolactin concentrations were also measured before induction of anesthesia and the third postoperative dose of HSK21542, and 24 h after the first postoperative dose of HSK21542. In stage 2, the plasma concentration of HSK21542 was measured before induction of anesthesia, before and immediately after the third postoperative dose of HSK21542, and 24 h after the first postoperative dose of HSK21542.

All clinically significant abnormal values from laboratory test, vital signs, and ECG values were reported as AEs. All AEs were coded with the Medical Dictionary for Regulatory Activities (MedDRA ver. 23.1) and grouped by system organ class (SOC) and preferred term (PT). The severity of all AEs was graded according to the Common Terminology Criteria for Adverse Events (CTCAE ver. 5.0). All AE summaries and analyses in this trial refer to treatment-emergent AEs (TEAEs), defined as all AEs occurring after the initial use of HSK21542/placebo until to the end of follow-up period.

Supplementary Appendix 3. Definition of the analysis set

Safety outcomes were analyzed in the safety set that included all randomized patients who had received at least 1 dose of HSK21542 or placebo and for whom data on post-dose safety evaluation were available. Efficacy endpoints were analyzed on the basis of the full analysis set (FAS), which was defined as all randomized patients who received at least 1 dose of HSK21542 or placebo, and had at least 1 available efficacy evaluation according to the intention-to-treat principle (ITT). Plasma concentration analyses were performed on the basis of the pharmacokinetics analysis set (PKS), including all randomized patients who received at least 1 dose of HSK21542 with at least 1 measurable concentration, and had no protocol deviations that may have affected the PK data. In addition, prolactin concentrations were measured in pharmacodynamics analysis set (PDS), included all randomized patients in stage 1 who received at least 1 dose of HSK21542 or placebo with at least 1 measurable prolactin concentration and who did not deviate from the protocol that may have affected the prolactin concentration.

Supplementary Table 1. Summary of drug-related treatment-emergent adverse events of enrolled patients in stage 1 and stage 2 (safety set)

Drug-related TEAEs, termed by preferred term, n (%)						
		HSK21542 group*				
**Stage 1**	**Placebo group (n = 12)**	**Group 1 (n = 12)**	**Group 2 (n = 12)**	**Group 3 (n = 12)**	**Group 4 (n = 12)**	**Total (n = 48)**
Nausea	1 (8.3)	0	1 (8.3)	0	0	1 (2.1)
Vomiting	1 (8.3)	1 (8.3)	2 (16.7)	1 (8.3)	0	4 (8.3)
Abdominal distension	0	1 (8.3)	0	0	0	1 (2.1)
Decreased free triiodothyronine	1 (8.3)	0	0	0	0	0
Pollakiuria	0	0	0	0	1 (8.3)	1 (2.1)
**Stage 2**	**Placebo group (n = 20)**	**HSK21542-0.5 µg/kg group (n = 20)**	**HSK21542-1.0 µg/kg group (n = 20)**			
Hypokalemia	1 (5.0)	0	2 (10.0)			
Decreased thyroid-stimulating hormone	0	0	1 (5.0)			
Hypoesthesia	0	0	1 (5.0)			
Paresthesia	1 (5.0)	0	0			

Note. For HSK21542 dose groups in stage 1: group 1: preoperative 0.4 µg/kg + 0.2 µg/kg at postoperative 0 h, 8 h and 16 h; group 2: preoperative 1.0 µg/kg + 0.5 µg/kg at postoperative 0 h, 8 h and 16 h; group 3: 0.5 µg/kg at postoperative 0 h, 8 h and 16 h; group 4: 1.0 µg/kg at postoperative 0 h, 8 h and 16 h. Stage 2: HSK21542-0.5 µg/kg or HSK21542-1.0 µg/kg were administered postoperatively at 0 h, 8 h and 16 h.

Abbreviations: TEAE, treatment-emergent adverse event

Supplementary Table 2. Efficacy outcomes of patients enrolled in stage 1 (FAS)

	Placebo group (n = 12)	HSK21542 group (n = 47)				*P*-value
		Group 1 (n = 11)	Group 2 (n = 12)	Group 3 (n = 12)	Group 4 (n = 12)	
$SPID_{0-12h}$ (scores × min)						0.147
Mean (SD)	293.8 (1,291.7)	-972.5 (1,334.7)	-177.6 (1,394.4)	-95.3 (866.7)	-621.3 (1,303.7)	
Median (range)	339.0 (-2,179-2,628)	-708.0 (-3,446-377)	-146.5 (-2,839-2,972)	14.5 (-1,796-1,149)	-654.0 (-2,004-2,234)	
$SPID_{0-24h}$ (scores × min)						0.155
Mean (SD)	127.5 (2387.2)	-2487.8 (2930.0)	-695.1 (2749.8)	-620.8 (1948.1)	-1,394.8 (2,536.3)	
Median (range)	589.0 (-4339-3903)	-1464.0 (-7539-472)	-1208.5 (-5719-5130)	-595.0 (-3923-2486)	-845.5 (-4,188-3,971)	
PID (scores × min)						
LSMD vs. placebo (95% CI)		0.34 (-0.59, 1.26)	0.37 (-0.51, 1.24)	0.45 (-0.35, 1.26)	0.55 (-0.46, 1.57)	
P-value vs. placebo*		0.459	0.391	0.253	0.272	
Patients administered with morphine 0-12 h after first dosing of the experimental drugs, n (%)	4 (33.3)	5 (45.5)	3 (25.0)	4 (33.3)	2 (16.7)	0.657
Cumulative dosage of morphine 0-12 h after first dosing of the experimental drugs (mg)						0.492
Mean (SD)	1.0 (1.48)	4.0 (6.65)	1.1 (2.2)	1.0 (1.5)	0.8 (1.9)	
Median (range)	0.0 (0-3)	0.0 (0-22)	0.0 (0-7)	0.0 (0-3)	0.0 (0-6)	
Patients administered with morphine during 0-24 h after first postoperative dosing of the experimental drugs, n (%)	4 (33.3)	6 (54.5)	3 (25.0)	5 (41.7)	2 (16.7)	0.377
Cumulative dose of morphine 0-24 h after first postoperative dosing of the experimental drugs (mg)						0.382
Mean (SD)	1.8 (2.7)	4.3 (6.5)	1.6 (3.2)	1.3 (1.5)	1.3 (3.5)	
Median (range)	0.0 (0-6)	3.0 (0-22)	0.0 (0-10)	0.0 (0-3)	0.0 (0-12)	
Time of first injection of morphine (min)						0.164

Mean (SD)	256.1 (204.1)	148.1 (328.9)	127.9 (85.9)	350.8 (469.3)	384.5 (288.1)	
Median (range)	226.7 (61.3-509.8)	15.5 (6.7-819.4)	160.3 (30.6-192.9)	181.8 (61.3-1,179.9)	384.5 (180.8-588.2)	
Patients with NRS ≤ 3 during 0-12 h postoperatively administration, n (%)	7 (58.3)	5 (45.5)	7 (58.3)	8 (66.7)	8 (66.7)	0.861
Patients with NRS ≤ 3 during 0-24 h postoperatively administration, n (%)	7 (58.3)	4 (36.4)	7 (58.3)	7 (58.3)	8 (66.7)	0.692
Analgesia duration (min)						0.866
Mean (SD)	1,237.9 (288.5)	1,311.2 (184.1)	1,310.4 (205.7)	1,382.3 (101.0)	1,253.4 (432.9)	
Median (range)	1,412.9 (683.0-1,442.7)	1,382.0 (833.0-1,466.0)	1,413.7 (877.0-1,443.7)	1,426.6 (1,149.0-1,460.8)	1,431.7 (14.6-1,598.3)	
Satisfaction score for patients						0.098
Mean (SD)	8.6 (1.8)	9.2 (0.9)	9.3 (1.5)	9.9 (0.3)	9.5 (0.8)	
Median (range)	9.0 (5-10)	9.5 (8-10)	10.0 (5-10)	10.0 (9-10)	10.0 (8-10)	
Satisfaction score for clinician						0.733
Mean (SD)	8.9 (1.2)	9.4 (1.0)	9.2 (1.0)	9.3 (0.9)	8.9 (1.2)	
Median (range)	9.0 (7-10)	10.0 (7-10)	9.5 (7-10)	10.0 (8-10)	9.0 (6-10)	

Note. *Data were compared using mixed model repeated measures (MMRM) methods, with the baseline pain score, treatment, time point and interaction between treatment and time point as fixed effects, and patients as random effects.

For HSK21542 dose groups, group 1: preoperative 0.4 μg/kg + 0.2 μg/kg at postoperative 0 h, 8 h and 16 h; group 2: preoperative 1.0 μg/kg + 0.5 μg/kg at postoperative 0 h, 8 h and 16 h; group 3: 0.5 μg/kg at postoperative 0 h, 8 h and 16 h; group 4: 1.0 μg/kg at postoperative 0 h, 8 h and 16 h.

Abbreviations: ANOVA, analysis of variance AUC, area under curve; LSMD, least squares mean difference; NRS, numerical rating scale; PID, pain intensity difference; SD, standard deviation; $SPID_{0-12h}$, time-weighted summed pain intensity differences over 12 h; $SPID_{0-24h}$, time-weighted summed pain intensity differences over 24 h

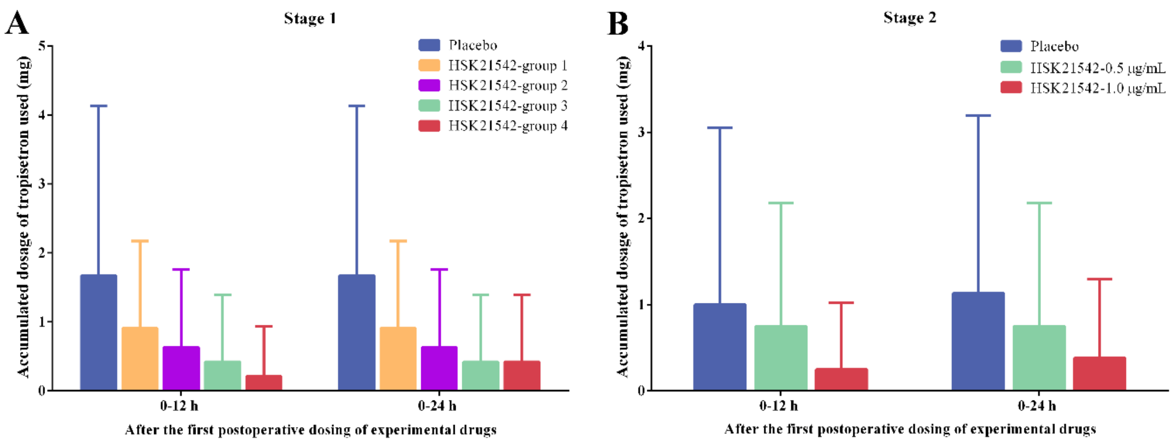

Supplementary Figure 1. The accumulated dosage of tropisetron after the first postoperative dosing of experimental drugs in (A) stage 1 and (B) stage 2

For HSK21542 dose groups in stage 1: group 1: preoperative 0.4 μg/kg + 0.2 μg/kg at postoperative 0 h, 8 h and 16 h; group 2: preoperative 1.0 μg/kg + 0.5 μg/kg at postoperative 0 h, 8 h and 16 h; group 3: 0.5 μg/kg at postoperative 0 h, 8 h and 16 h; group 4: 1.0 μg/kg at postoperative 0 h, 8 h and 16 h. Stage 2: HSK21542-0.5 μg/kg or HSK21542-1.0 μg/kg were administered at postoperative 0 h, 8 h and 16 h.

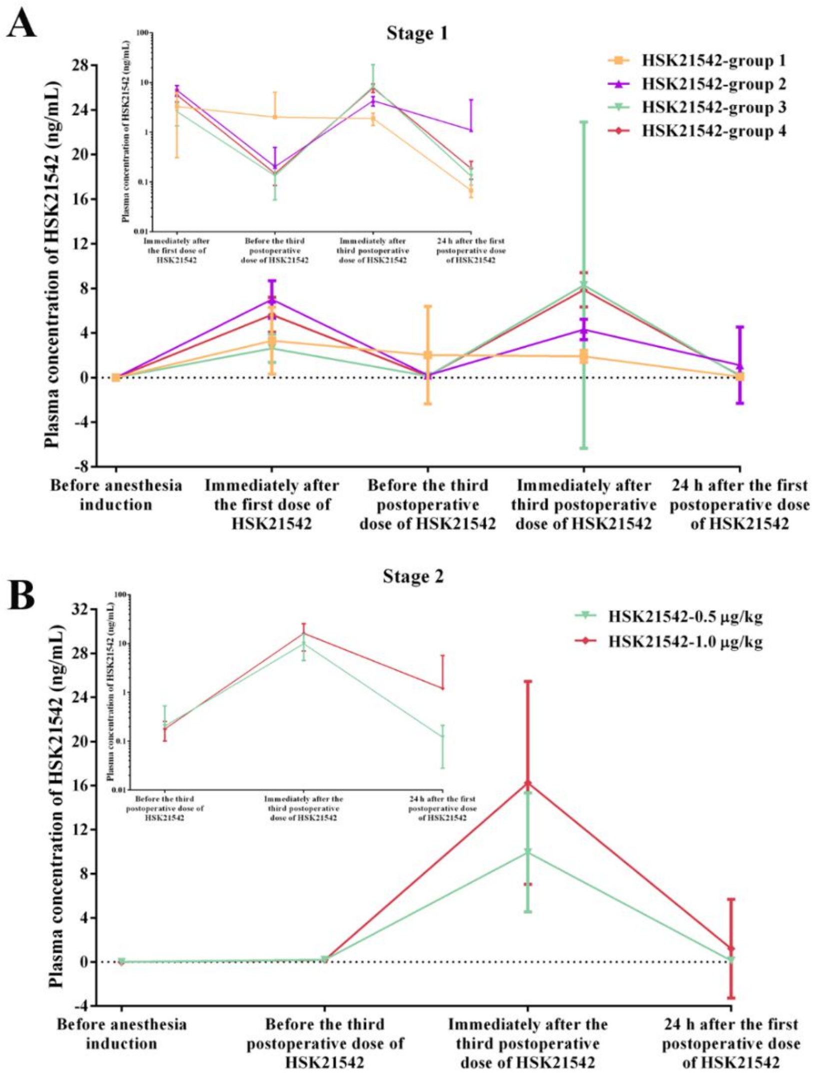

Supplementary Figure 2. The plasma concentration of HSK21542 in patients enrolled from (A) stage 1 and (B) stage 2 (linear and semi-log)

For HSK21542 dose groups in stage 1: group 1: preoperative 0.4 μg/kg + 0.2 μg/kg at postoperative 0 h, 8 h and 16 h; group 2: preoperative 1.0 μg/kg + 0.5 μg/kg at postoperative 0 h, 8 h and 16 h; group 3: 0.5 μg/kg at postoperative 0 h, 8 h and 16 h; group 4: 1.0 μg/kg at postoperative 0 h, 8 h and 16 h. Stage 2: HSK21542-0.5 μg/kg or HSK21542-1.0 μg/kg were administered postoperatively at 0 h, 8 h and 16 h.

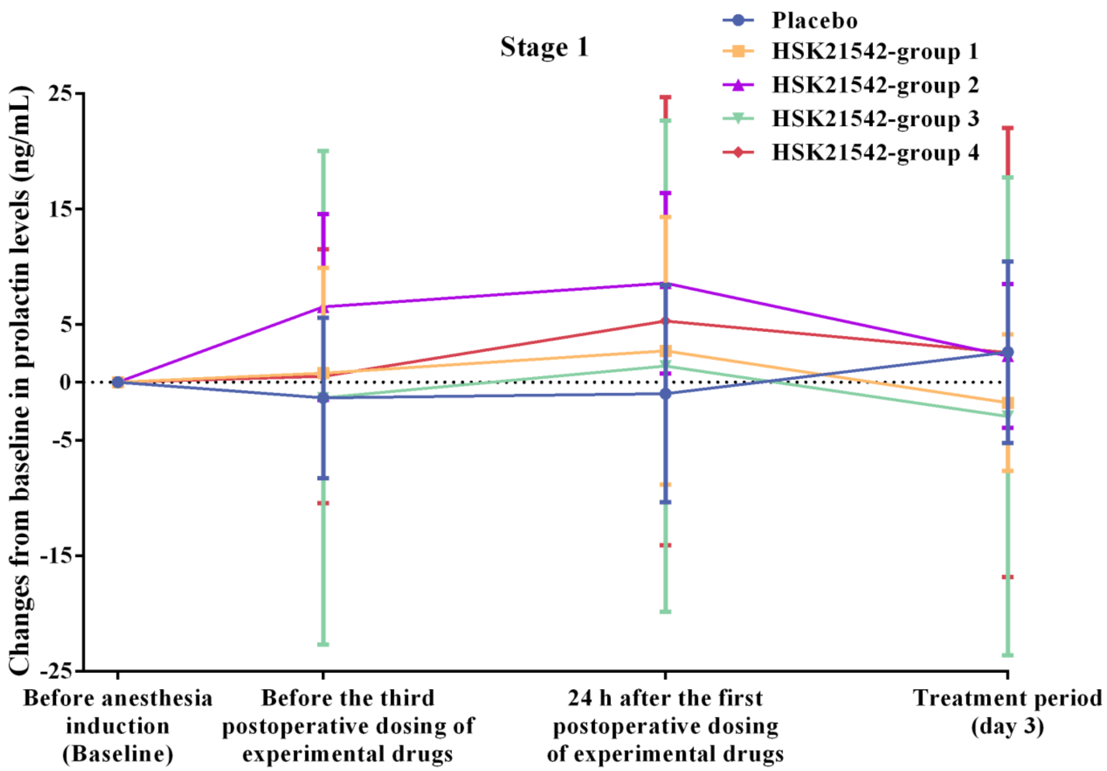

Supplementary Figure 3. Changes from baseline in prolactin concentrations of patients enrolled in stage 1

For HSK21542 dose groups, group 1: preoperative 0.4 μg/kg + 0.2 μg/kg at postoperative 0 h, 8 h and 16 h; group 2: preoperative 1.0 μg/kg + 0.5 μg/kg at postoperative 0 h, 8 h and 16 h; group 3: 0.5 μg/kg at postoperative 0 h, 8 h and 16 h; group 4: 1.0 μg/kg at postoperative 0 h, 8 h and 16 h.
